# Students experiences of an 8-week mindfulness-based intervention at a college of opportunity: a qualitative investigation of the mindfulness-based college program

**DOI:** 10.1186/s12889-022-14775-5

**Published:** 2022-12-13

**Authors:** William R. Nardi, Nour Elshabassi, Jayson Spas, Alex Zima, Frances Saadeh, Eric B. Loucks

**Affiliations:** 1 Department of Behavioral and Social Sciences, Brown University School of Public Health, Providence, RI USA; 2 Department of Psychology, Rhode Island College, Providence, RI USA; 3 Department of Epidemiology, Brown University School of Public Health, Providence, RI USA

**Keywords:** Mindfulness, Meditation, Qualitative research, Mental health, Undergraduate, COVID-19

## Abstract

**Background:**

Mindfulness-based programs have the potential to improve the well-being of undergraduate students by reducing anxiety, depression, and isolation in the wake of the COVID-19 pandemic. The aim of this qualitative study was to explore lived experiences of undergraduates in a mindfulness-based program at a “college of opportunity” that has high proportions of first-generation college students. Specifically, we sought to: (1) explore the application of mindfulness practices in students’ daily lives; (2) explore how participants believe mindfulness training affected their health and well-being; (3) learn participants’ recommendations and suggested changes for mindfulness-based interventions in future iterations.

**Methods:**

Students were recruited from XXX and consented to participate in semi-structured digitally conducting interviews after the completion of the 8-week intervention in the Fall/Spring 2020 academic year. Data were analyzed using Applied Thematic Analysis and a codebook was constructed using a consensus-driven process using both a priori and emergent coding. All transcripts were double-coded, and concordance was achieved for all interviews.

**Results:**

Qualitative results indicated that the most applied practices were those that could be easily incorporated into a daily routine. Furthermore, students reported an increased ability to cope with a variety of stressors, decreased reactivity, and enhanced resilience specifically concerning mental health challenges. Additionally, engaging in mindfulness training improved students’ ability to navigate social distancing measures, other COVID-19-specific stress, and enhanced motivation for self-care practices to sustain well-being. Concerning preferred intervention delivery modality, participants stated that due to multiple, growing responsibilities (e.g., pressures of commuting to class) they preferred in-person delivery, shorter sessions over a longer period, with classes in the morning or early afternoon.

**Conclusions:**

Findings provide pragmatic and psychosocial insights into students’ application of mindfulness training across personal, professional, and academic domains enhancing their well-being. This work builds on qualitative work concerning students’ perceptions and applications of mindfulness while offering insights into the future of mindfulness programs among undergraduates.

**Trial registration:**

Clinicaltrials.gov NCT03124446.

**Supplementary Information:**

The online version contains supplementary material available at 10.1186/s12889-022-14775-5.

## Background

Undergraduate students are at a critical stage in life marked by personal growth and professional opportunities as well as increased levels of stress, risk-taking behaviors, and maladaptive coping strategies, such as heavy alcohol and drug use [1–3]. In recent years, they have also reported significantly elevated levels of anxiety, depression, and loneliness compared to previous cohorts [2, 4–6]. Unfortunately the emergence of the COVID-19 pandemic exacerbated stress, anxiety, depression, isolation, and sedentariness due to social-distancing guidelines and strain on healthcare systems [7–9]. In a recent survey of 962 undergraduate students over 100 US universities, 73% reported mild depression, 63% mild anxiety while 15.2% stated they experienced severe anxiety and 23.4% reported severe depression an increase when compared to the US undergraduate student population in 2020 [10, 11]. With the increasing stress and mental health strain experienced over recent decades, they may need continued access to health care support to sustain well-being and maintain mental health [8, 9, 12].

Mindfulness Based Programs (MBPs) present a promising intervention for improving the well-being of undergraduate students [13–15]. *Mindfulness* has been defined as: (1) “the self-regulation of attention so that it is maintained on immediate experience,” and (2) “adopting a particular orientation toward one’s experiences in the present moment… characterized by curiosity, openness, and acceptance.” [16] MBPs designed to train university students in the practice and application of mindfulness skills have also been shown to significantly reduce anxiety and depression among undergraduate students [17–19]. Promisingly, multiple systematic reviews of quantitative and mixed methods trials have concluded that MBPs demonstrate initial effectiveness [15, 20]. A recent 2020 meta-analysis of 51 randomized controlled trials (RCTs) found that MBPs decreased distress, anxiety, depression, rumination and increased well-being compared to passive controls at follow-up ranging from three to twenty months across the studies [20]. However, studies were conducted among primarily upper-middle class, white, female students limiting their generalizability [13, 15]. In addition, there was a broad array of formats for the delivery of mindfulness courses including self-directed, digital delivery (i.e., mobile, online) as well as abbreviated MBPs and formal, evidence-based interventions (i.e., Mindfulness-Based Stress Reduction, Acceptance & Commitment Therapy) [20]. The findings from these recent reviews indicate (1) the field continues to requre more methodolically rigorous, evidence-based MBPs delivered among culturally, racial/ethnically, and socioeconomically diverse student populations; (2) comprehensive qualitative investigations of these students perceptions, application, and attitudes regarding mindfulness training are lacking [13, 14, 18, 20–23].

Although promising quantitative evidence exists regarding effects of MBPs for college students’ health, rigorous qualitative investigations of student’s experiences regarding mindfulness and health are lacking and will be critical as the field grows. Thorough qualitative investigations allow researchers to explore participants subjective experiences (e.g., thoughts, perceptions, values, feelings, etc.), investigate participants’ perceptions, identify patterns within individuals’ lived experiences, as well as develop hypotheses to inform future work and theoretical development. As a result, qualitative studies can not only enhance our understanding of student’s experiences and inform evidence-based customizations of interventions for future studies among unrepresented student populations, but also expand on quantitative work that has been completed. Unfortunately, qualitative work within this field remains limited. A recent systematic review identified only eighteen comprehensive qualitative investigations despite significant growth in research on MBPs among college students [23]. In addition, few studies have investigated the experiences of students in mindfulness training after the onset of the COVID-19 pandemic and after social distancing measures were implemented [24].

The Mindfulness-Based College (MBC) program is an evidence-informed adaptation of the Mindfulness-Based Stress Reduction (MBSR) program that was specifically designed for undergraduate students and has the potential to reach a wider undergraduate population [25]. MBC builds on a foundation of mindfulness skills (e.g., meditation, yoga, self-awareness, attention control, emotional regulation, etc.) through the use of a standardized curriculum. MBC directs participants to implement this training toward their relationship with several health-related factors relevant for young adults. In addition, this intervention also targets theoretical mechanisms to increase self-regulation, specifically self-awareness, emotional regulation, and attention control [26]. In an RCT (*n* = 96), MBC significantly improved depressive symptoms (*p* = 0.03), loneliness (*p* = 0.03), sleep quality (*p* = 0.04) and decreased sedentary activity (*p* = 0.006) compared to enhanced usual care control [25].

The present study qualitatively explored MBC with students from a primarily undergraduate institution that is considered a “college of opportunity” with 45% identified as first-generation college students (FGCS) [27]. FGCS are college students whose parents or guardians have not completed a bachelor’s or four-year college degree [28, 29]. Research has shown that nearly 1 in 6 college students are FGCS. Of FGCS, a disproportionate number are individuals from historically unrepresented groups including students primarily from low socioeconomic backgrounds as well as people of color [28–32]. Further, FGCS are more likely to be older, work full-time, and live off campus with commuter status compared with non-first-generation students. These differences may affect numerous health-related behaviors and increase life stress which continues to increase as a result of the COVID-19 pandemic [33]. Research has found that FGCS are significantly more likely to report depression and anxiety as well as experience financial hardships, abuse, food/housing insecurity and challenges adapting to online instruction [10, 11]. Although research indicates that FGCS demonstrate higher levels of academic and personal resilience and self-efficacy compared with non-FGCS there remains a paucity of research regarding how FGCS are managing pandemic related stress and what programs (e.g., MBPs, etc.) may be supportive during this time [34, 35].

The aim of this qualitative study was to explore lived experiences of undergraduates in a mindfulness-based program at a “college of opportunity” that has high proportions of first-generation college students. Specifically, we sought to: (1) explore the application of mindfulness practices in students’ daily lives; (2) explore how participants believe mindfulness training affected their health and well-being; (3) learn participants’ recommendations and suggested changes for mindfulness-based interventions in future iterations.

## Methods

### Participants

Participants (*n* = 16) were recruited from a community college in Rhode Island, United States, with a largely commuter student body (i.e., in 2019, 85% of undergraduate students were commuting) and a “college of opportunity” for first generation students. The college’s mission is to “offer accessible higher education of the finest quality to traditional and nontraditional students”. It is a community college that serves over 9000 students from Massachusetts, Connecticut, and Rhode Island. Participants were eligible for this study if they were: (1) 18-32 years of age; (2) Currently matriculated at the school; and (3) Able to read, write, and speak in English. Exclusion criteria were: (1) Current regular meditation practice (i.e., practicing meditation more than once a week); (2) Serious medical illness precluding regular class attendance; (3) Current substance abuse, suicidal ideation; and (4) History of bipolar or psychotic disorders or self-injurious behaviors.

### Recruitment

Brown University was the Institutional Review Board of record, approved all study procedures (#1608001570), and all research was conducted consistent with the Ethical Principles for Medical Research (i.e., Declaration of Helsinki). Participants were recruited for the study using a multi-method approach. Recruitment for the Fall 2019 session consisted of social media postings (e.g., Instagram, Facebook), flyers, and email recruitment and resulted in a very small class size (*n* = 4). Recruitment procedures were modified for the second iteration of the intervention based on feedback from students as well as expertise offered during discussions with faculty and administration. As a result, during enrollment for the second class offering (i.e., Spring 2020), study staff met with classes on the campus with the permission of the academic departments where they presented MBC to the students, answered questions about the intervention and provided study contact information (e.g., flyers) to interested students. This proactive recruitment procedure was facilitated by faculty support for the study. The partnership with specific faculty, as well as direct recruitment through speaking at invited classes, resulted in enrollment of 12 students for the second cohort for a total of 16 participants.

### The mindfulness-based college intervention

MBC is based on, and classroom time-matched to, the standardized MBSR intervention described in previous research [36]. Customizations of MBC include education on determinants of young adult well-being and mindfulness education and practices were designed to be implemented by students in the context of specific health behaviors: diet, mindfully engaging in physical activity and dietary change, awareness of alcohol use, stress, enhancing sleep, mindful social engagement, deep relaxation, and academic performance.

In addition, while MBSR recommends 45 minutes of formal mindfulness practice recordings at least 6 days a week, MBC is amended to allow participants to choose their home practice lengths, through 10-, 20-, 30- and 45-minute recordings. For additional details concerning the intervention as well as results from the preliminary acceptability and feasibility study please see Loucks et al. 2021 [25].

The MBC intervention was taught by an instructor who was not involved in data collection or analyses, who had extensive experience in offering MBPs among college students and to diverse populations, and was fluent in both Spanish and English. In addition, the instructor was a certified both through MBSR and MBC instructor trainings. MBSR teacher certification an extensive process of accreditation detailed elsewhere [37]. MBC instructor training involves: (1) An initial 40-hour online videoconference or in-person training where the entire MBC course is observed by all trainees; (2) Two half-day in-person training retreats where MBC-specific teaching modules are practiced in peer groups, supervised by the senior MBC trainer, using peer- and trainer-provided feedback; and, (3) Supervised teaching of MBC in non-study participants is done until quality is established, documented by at least 90% mean adherence to the MBC curriculum guide.

### Interview procedures

All sixteen students that completed the intervention were invited to return for interviews. After the intervention concluded, participants were contacted via email for the qualitative research, the only inclusion was that they had been part of the MBC intervention at Rhode Island College. All interviews were conducted via Zoom teleconferencing and participants scheduled a time with research staff via email. This procedure was revised to comply with all the social-distancing and health guidelines during the COVID-19 pandemic. Prior to the interview, participants were sent: (1) a copy of the consent form for their review, and (2) an outline of all 9 sessions with course content as a reminder of all the work covered in the course. Since students may have been in their home or a private space prior to the interview, the study personnel notified them that they would not be required to have their camera on if they preferred to maintain their privacy. All participants elected to have their cameras off during the interview. Participants were asked to verbally consent to the interview as well as to being recorded and consent was documented in the interview transcripts. Participants were compensated 50 USD for participating in the interview.

### Interview guide

The semi-structured interview guide was modified from our previous qualitative work [38]. Specifically, in this study, the session began with an overview of the main activities in each class to refresh student’s memories of all the course content (see Supplementary material file 1). From there, participants were asked a series of questions relevant to the study aims including, “What was most helpful for you about this course?”, “After going through this mindfulness intervention, what is your understanding of how it may improve your well-being?”, and “We want to make this intervention better. You have been through it once. How do you think we can make it better?”. A full copy of the interview guide is available in Supplementary material file 1.

Participants were also asked specific questions concerning intervention delivery to inform future implementation. These questions included: “One question we have is whether it would be easier for students to attend two shorter (e.g., 1.5 hour) courses over the week as opposed to 1 longer course. What are your thoughts on this?”, “What are your thoughts on digital delivery of this intervention?”. As a note, during cohort 2 (*n* = 12) due to the COVID-19 pandemic the intervention was approved by the Brown IRB to shift to digital delivery starting at week 5 of the intervention. This presented a unique opportunity to also gain insight from students who had experienced both an in-person and digitally delivered MBP.

### Data analysis

All interviews were conducted via Zoom teleconferencing with automatic transcription services. Interview transcripts were de-identified, reviewed for accuracy and analyzed using NVIVO 12. Videoconference in-depth interviews (IDI) were chosen to lower participant burden and increase participation rates, especially when considering the health and safety concerns regarding the COVID-19 pandemic [39, 40]. The interviews lasted 20-45 minutes and were conducted by a trained facilitator (WN) a cisgendered male PhD candidate with previous qualitative facilitation experience with no other research team members present in interviews [25, 41, 42]. The facilitator handled communication for the research study and the participants were familiar with him prior to the interviews. In addition, participants were aware that the facilitator was a part of the study notified that the facilitator has been involved in multiple mindfulness research studies with this study team [25, 41, 42]. The facilitator did not elaborate on additional characteristics (e.g., reasons and interest in the topic, goals, etc.) and kept descriptions of his role to within the context of the interview (e.g., here to listen, non-judgmental, respect for all feedback, etc.). Field notes were taken during interviews and informed the development of the initial coding structure.

Data were analyzed using Applied Thematic Analysis (ATA) [43]. ATA is a inductive approach consisting of clear analytic guidelines designed to enhance research credibility and was deemed appropriate given the scope of the research (e.g., application of skills training rather than theory development) [43]. ATA involves a rigorous process of identifying and examining interview passages as a function of research questions, documentation and transparency in the data reduction process, and guidance for querying coding repetitions across participants or identifying distinct coding between participant narratives [43].

Zoom transcription services were used for the initial transcript. All transcripts were reviewed by WN and NE and compared to the original audio files. All transcripts were reviewed to remove participant identifiers, transcriptions errors and indiscernible parts of recordings.

The initial coding structure was based on the facilitation guide as well as field notes from the facilitator. The initial codebook was then refined (see Supplementary material file 2) using a consensus-driven process which consisted of both a priori and emergent coding conducted in NVIVO 12 (QSR International, Doncaster, Australia). First, both members of the research team (WN, NE) coded 20% of the interviews (*n* = 4). Open coding and analytic memos were used to document potential relationships, emergent coding, as well as note patterns in the data. Coders then met to discuss memos and preform conflict resolution of the preliminary coding. After the discussion, a finalized coding structure with coding definitions was established. Coders double coded all remaining interviews (*n* = 11) using the coding structure. Discrepancies in coding were resolved through line by line review of all transcripts (*n* = 15) [43]. As a result, inter-coder agreement (i.e., concordance) was > 85% for all interviews. Documentation for all decisions regarding the codebook development, resolution of conflicts, and consolidation of emergent codes were logged using a digital audit trail in the NVIVO project.

As coding was underway, the data reduction process involved refining and developing emerging themes using systematic comparisons of participants experiences through a series of coding comparison queries (i.e., differences in the proportion of references for each code between FGCS and non-FGCS), quantitative review of references per code and codes per participant to identify most prevalent coding and a series of three team discussions which included the advisement of a content expert (EL) to review coding and inform theme development and refinement. As fifteen of sixteen participants elected to complete the interviews data saturation was deemed to be met due to almost all participant being interviewed (i.e., no new information available from sample) as well as consensus on no new coding/themes in coding meetings, consistent with suggested practice [44]. Data refinement was conducted to investigate each research aim (e.g., exploring how mindfulness practices were used, understanding any relationship between mindfulness and health, investigating how to enhance the program, etc.) and the results section is presented with aims and the refined themes.

## Results

### Participants/demographics

Interviews were completed within 2 months of intervention completion during April and May 2020 at the onset of the COVID-19 pandemic. The window to interview was initially 1-month post program however, the pandemic and adjusting to social distances measures presented scheduling difficulties (e.g., course shifts, childcare/family responsibilities, etc.) which limited the availability of participants. There was a 94% participation rate (*n* = 15) with one participant electing not to complete the post-intervention interview despite three contact attempts by research staff. The racial and ethnic composition of the group is shown in Table 1. Six (42%) of the participants indicated that they were first generation college students. Over half the sample was female (*n* = 12; 75%) and identified their sexual orientation as “straight”. Most students attended more than 5 class sessions. However, completion rates in the second group (*n* = 12) averaged 6 sessions with several decreasing class attendance after the shift to online delivery. In addition, students were not restricted to a program, so the sample was mixed with students concentrating in various departments including educational studies (e.g., special education, health and physical education, nursing, arts and sciences (e.g., biology, music, etc.), and business. Of the 15 participants, one elected not to complete the demographic questionnaire as such baseline characteristics are based on 14 participants.

**Table 1:** Student baseline demographics

	^a^Overall (*n* = 14)
**Mean Age, years**	24.8 (4.03)
**Current Gender Identity**^a^	
Female	11 (78.6%)
Male	3 (21.4%)
**Race/Ethnicity**^a^	
African American/Black	2 (14.3%)
Cape Verde	1 (7.1%)
Caucasian/White	8 (57.1%)
Latino/Hispanic	2 (14.3%)
Southeast Asian	1 (7.1%)
**Sexual Orientation**^a^	
Bisexual	1 (7.1%)
Gay	3 (21.4%)
Straight	10 (71.4%)
**Academic Year**^a^	
1	3 (21.4%)
2	1 (7.1%)
3	4 (28.6%)
4	2 (14.3%)
5	4 (28.6%)
**First Generation Student**^a^	
Yes	6 (42.9%)
No	7 (50.0%)
N/A	1 (7.1%)

^a^One student did not complete the demographic questionnaire and was unable to be recontacted for the study. Marked baseline statistics are thus based on *n* = 14

In reviewing the FGCS transcripts (*n* = 6) compared with the non-FGCS (*n* = 7), we found no notable differences thematically when reviewing participants’ descriptions of their experiences while in the mindfulness program. In addition, we reviewed the proportions of coding frequencies across all themes between those who were FGCS and non-FGCS. We found no proportional differences in coding that might indicate FGCS status affected uptake of mindfulness training and application of the mindfulness learned in the program. It should be noted this lack of differences in coding and experiences could be a limitation of the interview guide and has been addressed below in the limitations.

Themes derived from all participants experiences are presented under their associated research aim (1) exploring the application of mindfulness practices in daily life; (2) understanding how participants believe mindfulness training affected their health and well-being; (3) learn participants’ recommendations and suggested changes for future mindfulness interventions. For aims, resulting themes and example quotations for each theme, please see Table 2.

**Table 2:** Qualitative themes and sub-themes with representative quotations

Aims & Themes	Codes	Example Quotes
***Aim 1:*** *Explore the Application of Mindfulness Practices in Students’ Daily Lives*		
***Theme 1.*** *The most used practices are those that could be integrated into daily life.*	*Mindfulness practices outside of class* *Mindful Walking* *Mindful Eating*	*“I’m not used to just like to stop and be mindful while I’m eating. I just like go through and eat it as quick as possible to eat something. So, I can now acknowledge that I can, like, take the time enjoy the food.” (P756, male)* *“I feel like most days you just walk, to walk, you have to get to one place or you’re going somewhere you’re talking so you don’t really like think about the actual movement and break it down. So, kind of just like taking yourself…like to slow down. I think that that was really cool and just something that like I guess I’ve never really focused on before.” (P540, female)*
***Theme 2.*** *Students often applied mindfulness to existing behaviors and activities.*	*Mindfulness enhances existing routine* *Daily Practice*	*“It [the program] made me realize that you can mindfully do things, even when you’re working, and I found that very calming. And it helped me not to be so stressed, so like mindfully brushing my teeth every morning and every night. It helps me realize and be grateful for the things around me as well.” (P213, female)* *“Even when I was like in the shower I’d be like “Ok I’m washing my hair” I would say like talk to myself out loud like “Ok I’m washing my hair, I’m washing my body, I’m shaving my legs, I’m shaving my right leg, I’m shaving my left leg”, like go through everything, and … just like really pay attention to what I was doing.” (003, female)*
***Theme 3.*** *Mindfulness increased students’ engagement activities motivated by self-care.*	*Engaging in self-care*	*“It really emphasized or made me realize that like I actually need to take care of my mental well-being. Because, like, a few months ago, or like last year coming into this year I had that mentality that mental health does not exist if I’m physically well then, I’m good. But taking this course and made me realize that in order for me to be even more productive I need to be mentally well as well and I need to set boundaries.” (P213, female)*
***Aim 2:*** *Explore how participants believe mindfulness training affected their health and well-being*		
***Theme 1.*** *Mindfulness was used most often to cope with an array of stressors.*	*Overall stress management* *Stressful living situation* *Anxiety* *Self-kindness*	*“I use it during the exam actually…final exams. You know, it’s a stressful time and me personally, I’m the kind of person to study. A lot. And then when the test comes, I forget things, but before one of my exams. I just did like a deep breathing exercise and it kind of helped me to just boost my confidence and just relax.” (P203, female)* *“This was my first time commuting from school so that was a really hard experience for me just because I don’t have the best home life and for a while I kind of just tried to push that in the back of my mind. But rough these meditations I actually kind of reached a point where I acknowledged just how unstable I felt living at home. And that was something that through the mindfulness courses I just kind of acknowledged and I realized that I needed to take mindful action.” (001, female)*
***Theme 2.*** *Mindfulness practices help to handle COVID-19 specific stress.*	*COVID-19 related stress*	*“Probably just with school and work because it’s been like really hard doing everything online. But just knowing that I can use this practice as a way to help coach has really helped me get through day-to-day tasks and anything pertaining to schoolwork.” (P533, female)* *“I just started working at a hospital. So, there are times where it can get really hectic. A lot of things going on at once and breathing exercises just helped me to calm down in the moment and just, you know, handle whatever it is that’s going on at the time. That’s one of the most recent things that I’ve been doing.” (P203, female)*
***Theme 3.*** *Mindfulness enhanced stress coping by increasing the ability to acceptance of the present moment and let go of the experience to move forward.*	*Letting go* *Acceptance* *Lower self-criticism* *Non-judgment*	*“I used to just like “oh my gosh” and just shut down and cry or have an anxiety attack and now I’m like genuinely “it’s out of my control” I’m going to do what I can do and… like… I don’t even freak out anymore which is amazing. So, my anxiety levels have definitely gone down.” (002, female)* *“I realized that I need to accept what is. And I need to take mindful action in order to you know, feel more secure with myself. And there were multiple instances where I had certain things arise where I realized that I just need to think upon and rather than just feel like anxiety I decided to take more of a mindful approach to it. In my situation. (001, female)*
***Theme 4.*** *Students also believe mindfulness enhanced resilience, especially for those with existing mental health conditions.*	*Resilience* *Mindfulness enhances recovery* *Increased patience*	*“I had a depression like screening and conference call with my primary care. And I’ve had the past two anti-depressants that I’ve been on I’ve been allergic to, and even with that she’s even like she even noticed today. She’s like, you see much more chipper and things like that. So, I feel like I have like a more positive outlook on things, and I don’t let everything else going on around me that I can’t really control, control me anymore.” (P344, female)* *“So, if I’m going into a sit-down meal already really anxious because I have anorexia so, I’m like well into my recovery now, or trying to be, but if I go into a meal and I’m already stressed out about it, like freaking out internally and it’s a challenge meal those are super highly stressful situations for me. And just breathing and like even stopping to think back and like meditating to get through it has helped me a lot.” (P002, female)*
***Theme 5.*** *Mindfulness was critical for grounding themselves during difficult experiences as well as enhancing the value of positive experiences.*	*Grounding* *Mind-Body awareness* *Gratitude*	*“So just moving forward I think this course is super helpful and just being able to acknowledge what is and acknowledging when you’ve put in all your efforts and you just have to do some more self-work, self-reflection and just to put that energy into just grounding yourself through mindfulness.” (001, female)* *“I’m learning to recognize, you know, like maybe anxiousness or overwhelmed-ness but being able to channel into deep breathing or focusing on the moment, the present moment, whether that be, you know, noticing or looking around and seeing five things you see or things you hear, you know, using your senses to get in touch with where you are at that time, that’s been really helpful to me to calm my anxiousness.” (206, female)*
***Aim 3:*** *Recommendations for Future Mindfulness Interventions*		
***Theme 1.*** *Social support is generated during in-person meetings so in-person more preferred method of delivery.*	*Class critiques* *Instructor comments*	*“To be able to go to a community where I can like get other insights and opinions and like share the experience with other people. So, I don’t think that the, I don’t think that doing it online would be very effective. Just because you don’t really get that personal like interaction you know. Like you don’t get that sense of community.” (003, female)* *To give a specific example. The meditation aspect. In person* versus *when we were digital. It was just easier to focus when we’re all together in a quiet room, um, you know, that may not necessarily be accessible to someone at home over Zoom, I guess. (P206, female)*
***Theme 2.*** *A split class format would fit into schedules more effectively but could be difficult for those who commute.*	*Course structure and delivery*	*“It just allows for people to attend rather than say we have a 2.5-hour class where that may cut into someone’s time elsewhere or like another class or like an appointment or just different things that people can have going on in a two-and-a-half-hour gap, rather than a one-and-a-half-hour gap.” (P206, female)* *“If it were at the beginning and at the end of the week it’s kind of would allow for more reflection and it’s sometimes easy to forget like if you’re doing like the reflections in the workbook or like logging certain things like we had to do throughout the program that was kind of difficult because a week is a long time. And it’s easy for me to forget a lot of things that I did. But if it were twice a week that would be easier.” (002, female)*

### Exploring the application of mindfulness practices in students’ daily lives

#### Theme 1. The most used practices are those that could be integrated into daily life

Although students were taught formal meditation practices (e.g., sitting meditations, body scans, etc.) during class, these were not the most used mindfulness trainings. The practices that they applied the most were mindfulness activities that could be easily incorporated into their daily routines.

Mindfulness of eating was introduced formally at the beginning of the intervention and was one of the most incorporated practices mentioned by students (*n* = 9). Participants described that eating mindfully allowed them to settle into the moment, fully experience the taste and sensations of the food, and choose to make healthier choices because they were becoming aware of the impact of diet on health.

I’m not used to just like to stop and be mindful while I’m eating. I just like go through and eat it as quick as possible to eat something. So, I can now acknowledge that I can, like, take the time enjoy the food. (P756, male).

Also, eight of the 15 participants discussed how the introduction to mindfulness of physical activity, both low intensity and higher aerobic activity, was a practice they had regularly incorporated. The most common application was mindful walking throughout the day. For example, going from class to class on campus as well as walking in their homes, neighborhoods, or local community when classes were shifted during the pandemic.

I feel like most days you just walk, to walk, you have to get to one place or you’re going somewhere you’re talking so you don’t really like think about the actual movement and break it down. So, kind of just like taking yourself…like to slow down. I think that that was really cool and just something that like I guess I’ve never really focused on before. (P540, female).

Several participants (*n* = 4) said that they added mindfulness to exercise routines they had prior to starting the course. They were applying the instruction offered during the class on mindfulness and aerobic activity as a component of their existing exercise routine and noticing that these activities can be enhanced with mindfulness.

I found the physical part very helpful because I even discovered on my own, you know recently that working out and exercising can really play a big role in mindfulness. (P533, female).

For one participant the class allowed her to modify her existing excise routine not only to incorporate mindfulness but to accommodate the restriction that resulted from the pandemic.

Physical activity and, and just now with this whole COVID thing and like not being able to go to the gym and the lack of exercising I focused on physical activity. Because I have not been able to do it so often as I would like to because of not being able to go to the gym. So just finding different methods, like going around my neighborhood, and going for a jog, you know, or either like walking my dog or you know those aspects to just try to like motivate me to like keep going you know keep focusing on physical activity. (P176, Female).

#### Theme 2. Students often applied mindfulness to existing behaviors and activities

In addition to adding new practices introduced during class to their lives students also described applying mindfulness to existing parts of their daily routine that were not formally discussed in class, transforming these activities into informal mindfulness practices. There was substantial variety in the behaviors that mindfulness was incorporated into, but studying was one of the more consistently mentioned. Three students explained how they had applied mindfulness during studying. As a result, they stayed more focused for longer periods of time, noticed earlier on when distracted, and were able to refocus when engaging with difficult material.

If I’m doing a reading for like one of my other like normal classes and it’s some research paper and it’s not super exciting like just reading that and focusing on the actual like words as well as sort of the bigger picture like content, like, what has this paper said so far, like in the last three pages. And then just sort of notice when my mind drifts off for like one sentence brings up something it makes me think of something else, just like notice that, and then come back to the reading. (P433, male).

In addition, individual students reported transforming a variety of other routine activities. Showering, goal setting, and listening to music were behaviors they began to engage in mindfully. These students indicated incorporating mindfulness into aspects of their hygiene routines (e.g., brushing their teeth, shaving), or taking short pauses in routine moments on campus (e.g., drinking coffee, sitting in the sun), enable them become more present in their moment-by-moment experiences.

It [the program] made me realize that you can mindfully do things, even when you’re working and I found that very calming. And it helped me not to be so stressed, so like mindfully brushing my teeth every morning and every night. It helps me realize and be grateful for the things around me as well. (P213, female).

#### Theme 3. Mindfulness increased students’ engagement in activities motivated by self-care

Over half (*n* = 10) of students mentioned that they shifted to intentionally engaging in behaviors that were motivated by self-care. For some students this meant choosing to eat healthier food to be kind to themselves (*n* = 4), engaging in physical activity to improve their health (*n* = 3), or pausing to take a break during busy days. Some described taking breaks from academic stress to rest with the intention of taking care of themselves, which then allowed them to study more effectively.

Being a college student and then going through all those new environmental changes and adaptations that you have to make regarding academic work. I think that implementing meditation and just deep breathing and taking time out of your week and I tried to actually do it daily, once I started doing this class. I tried to do at least just some like deep breathing and have some time for like quiet time just for myself, to kind of distress and like break up my weekend and reset. (P540, female).

One student described a significant shift in her perspective about self-care as a result of taking MBC. Prior to the course, she believed that mental health was not a component of well-being and prioritized only physical health. After taking the course she shifted her perspective and started attending to both her mental and physical health.

It really emphasized or made me realize that like I actually need to take care of my mental well-being. Because, like, a few months ago, or like last year coming into this year I had that mentality that mental health does not exist if I’m physically well then I’m good. But taking this course and made me realize that in order for me to be even more productive I need to be mentally well as well and I need to set boundaries. (P213, female).

### Understanding how students believe mindfulness training affected their health and well-being

#### Theme 1. Mindfulness was used most often to cope with an array of stressors

All of the students indicated that they used the mindfulness skills they’d learned to manage some type of life stress however, the specific stressors varied somewhat by student. Not surprisingly, academic stress was the most prevalent (*n* = 6). Students described how they became aware that they were stressed, for example studying or taking an exam, and tried to apply practices taught in class.

Like to just be able to be in the present has enabled me to you know stop looking at the past, stop worrying about the future, stop stressing out. Just to be more focus oriented. I had some exams coming up and like being able to only focus on studying and doing it mindfully has enabled me to improve my GPA and to be also able to have more control over my emotions. (P004, female).

I use it during the exam actually…final exams. You know, it’s a stressful time and me personally, I’m the kind of person to study. A lot. And then when the test comes, I forget things, but before one of my exams I just did like a deep breathing exercise and it kind of helped me to just boost my confidence and just relax. (P203, female).

Others (*n* = 4) explained that they were balancing academics as well as job-related stress. Mindfulness helped them become more aware of a difficult situation and take moments of self-care. For some participants, this involved taking a quick moment to breathe, away from sources of stress. For other participants, mindfulness practice allowed them to recognize when they needed to take more sustained action (e.g., set work boundaries). One student found that she was overworking herself responding to emails far past the point of when was healthy for her, even beyond expected working hours. The course allowed her to see the need to set boundaries and act to protect her health and well-being.

I’m supposed to work from a certain time period. But if people are emailing me at like 11 PM before I would like respond right away. But I realized that “No, I don’t think that’s healthy” working till like 11 or 12 and I can respond the next day, and that’s why: because that time is for me and I need to restore and reset. […] So this course it made me realize that you can mindfully do things, even when you’re working and I found that very calming. (213, female).

For a few (*n* = 2), commuting to class was stressful. Using the practices from class they recognized the stress and either modified their commute for less driving or took other actions. In one example a student described how it was her first year living off campus and commuting was a difficult experience. The course helped her recognize her situation and take action, mindfully making plans to modify her living arrangements in the future.

This was my first time commuting from school so that was a really hard experience for me just because I don’t have the best home life and for a while I kind of just tried to push that in the back of my mind. But through these meditations I actually kind of reached a point where I acknowledged just how unstable I felt living at home. And that was something that through the mindfulness courses I just kind of acknowledged and I realized that I needed to take mindful action. (001, female).

#### Theme 2. Mindfulness practices help to handle COVID-19 specific stress

Eleven of the 15 students interviewed participated in MBC during the onset of the COVID-19 pandemic. Of those, 6 students described that the course helped them to work with pandemic-related stress. For two students who were working in healthcare, the practices provided a space of relief in highly stressful situations.

I work in healthcare. So work is really stressful overall, there’s just probably not a day I don’t feel stressed after work and tense because there’s just so much going on and everything is so chaotic. So that [mindfulness practices] would be like a way I just try to use those techniques to help deal with stress and anxiety from like after work or from, like, a fight with like a friend or like a significant partner or like a breakup, like it’s just it’s good to learn about these things to help you… your own self deal with things. (P832).

For others (*n* = 3) the shift to online delivery of all classes was a difficult transition. They explained that staring at a computer screen for hours as well as being back home, away from social support, while taking classes was challenging. The meditations provided some relief helping them get through the day.

It’s been like really hard doing everything online. But just knowing that I can use this [mindfulness] practice as a way to help cope has really helped me get through day-to-day tasks and anything pertaining to schoolwork. (P533, female).

One student was working from home as a peer mentor as well as taking classes. Not only did she use the practices to help manage the stress, but she also became aware when being online was too much for her. Instead of trying to work through this she took appropriate action and, because of insight gained from the course, was able to recognize her limitations responding effectively.

I’ve had a lot of concussions in my life. So, looking at a computer screen constantly was becoming a severe issue for me. So, where I was looking at the computer from probably nine o’clock in the morning till nine o’clock at night and getting maybe a half hour break in between and it’s something that I couldn’t really control at that point. That’s what life had done. I contacted like Disability Services and explained and was able to modify some of my assignments where my professors would have to send me hard copies, rather than taking the quiz online. I would have to take the quiz; they would send me the hard copy and things like that. So that allowed me to like kind of take the step back. (P344, female).

#### Theme 3. Mindfulness enhanced stress coping by increasing the ability to acceptance of the present moment and let go of the experience to move forward

Ten students detailed how important mentally ‘letting go’ was especially when confronted with difficulty. More specifically, narratives from students describe a process of becoming willing to engage with difficulty, using grounding practices to steady themselves in the moment and choosing to let go when they recognized that situations were beyond their control. This process seemed to involve letting go of their attempts at controlling their experiences, specifically thoughts of anxiety and rumination (*n* = 7), as well as social challenges (*n* = 4), circumstances surrounding COVID (*n* = 4), and work stress (*n* = 2).

I used to just like “oh my gosh” and just shut down and cry or have an anxiety attack and now I’m like genuinely “it’s out of my control” I’m going to do what I can do and… like… I don’t even freak out anymore which is amazing. So, my anxiety levels have definitely gone down. (002, female).

Some students detailed letting go as an immediate action once they became aware of the difficulties but for eight students, acceptance of their circumstances was suggested as an important step before they were able to let go. Mindfulness practices allowed them to more objectively look at difficulties, and instead of resisting, they began to understand and accept both pleasant and difficult experiences will occur in life, thereby making it easier to let go. Some participants explained how they accepted their emotional experiences, others detailed accepting a difficult job situation and moving on, and others accepted past choices, thereby reducing judgment of themselves.

That was one situation where I realized that I need to accept what is. And I need to take mindful action in order to you know, feel more secure with myself. And there were multiple instances where I had certain things arise where I realized that I just need to think upon and rather than just feel like anxiety I decided to take more of a mindful approach to it. In my situation. (001, female).

#### Theme 4. Students also believe mindfulness enhanced resilience, especially for those with existing mental health conditions

Several (*n* = 9) students described a process of adapting to adverse circumstances over time coded as ‘resilience’. They described a series of adverse circumstances from difficult housing situations, COVID-19 isolation and academic difficulties, interpersonal stress, and job-related stress. They understood that these situations would continue to be sources of stress over time and that, through mindfulness practice, they could acknowledge the situation, take action to limit the effects of the stress (e.g., mindful action, breathing meditation), and manage the difficulty more effectively the next time they were confronted with this challenge. Importantly, students were able to be more objective using mindfulness to look clearly at a situation knowing that difficulties will eventually end which made the life circumstances easier to work with. Other students explained that they let go of sources of constant stress more easily knowing that the situation was out of their control and would continue for the time being.

It was one of those things where I acknowledged that I would have to go through this for longer. And sometimes it’s hard especially when you acknowledge something that doesn’t feel comfortable because then you still have to kind of live through it. And sometimes it’s just easier to not think about it. But because I was mindful because I was aware that I had to live on campus for the rest of the semester, but I did take the action knowing that my future can be changed. And that it doesn’t have to be permanent. (P001, female).

Three students in treatment with clinically diagnosed mental health conditions (anxiety, depression, and anorexia), described how mindfulness also enhanced their recovery and increased their resilience for managing adversity. All three students indicated that they came to the intervention hoping to enhance their ongoing treatment. Through the class they were able to recognize their thoughts and emotions regarding triggering situations, continue to engage instead of avoiding, and experienced the fading of difficult thoughts and emotions.

And just breathing and like even stopping to think back and like meditating to get through it has helped me a lot. Like before I would just freak out. I would shut down and I would just feel so defeated and have no confidence in myself. And now I kind of just allow myself to be here and be present and it’s ok to feel all of those things but like there are ways that I have now. (P002, female).

I had a depression like screening and conference call with my primary care. And I’ve had the past two anti-depressants that I’ve been on I’ve been allergic to, and even with that she’s even like she even noticed today. She’s like, you see much more chipper and things like that. So I feel like I have like a more positive outlook on things and I don’t let everything else going on around me that I can’t really control, control me anymore. I kind of take a step back and look at things from a different perspective and just try to stay in the moment and breathe and like I said, like if I can’t control it there’s no use really and stressing about it. (P344, female).

#### Theme 5. Mindfulness was critical for grounding themselves during difficult experiences as well as enhancing the value of positive experiences

Every student described the importance of becoming more aware of their thoughts, emotions, and physical sensations. Present moment awareness reportedly offered two primary functions: as an initial step in working with difficult life experiences and decreasing distractions increasing enjoyment of pleasant experiences.

Grounding during difficulty was the most common application for present moment awareness (*n* = 15). Multiple accounts explain that stress management was facilitated by becoming aware of thoughts, feelings and emotions in the moment which then allowed a choice of how to engage. Most (*n* = 11) reported that they chose to focus on their breathing or body sensation staying present with an unwanted experience and choosing how they would like to respond. Some (*n* = 2) described grounding by looking at objects in their immediate vicinity during a difficult experience, and one indicated that she would write down her thoughts and experiences instead of being “controlled by my thoughts or by the environment” (004, female).

I’m learning to recognize, you know, like maybe anxiousness or overwhelmed-ness but being able to channel into deep breathing or focusing on the moment, the present moment, whether that be, you know, noticing or looking around and seeing five things you see or things you hear, you know, using your senses to get in touch with where you are at that time, that’s been really helpful to me to calm my anxiousness. (206, female).

In other descriptions, students (*n* = 6) explained that by becoming more aware of the present moment they could engage more actively in positive health behaviors without being distracted, which increased the perceived value of the experiences. Some reported that they were able to focus more on academic tasks and performed better. For others, increased attention to the present moment made activities like eating, looking at something pleasant, or walking on a beautiful day, become more enjoyable.

Things are more enjoyable when I focus on them. And I also do them better. (P466, male).

Being aware of the small things in life. We usually don’t pay attention. So even sensations or sounds there’s something there we can be aware of. So, they’re like really important. So now when I go outside and it’s sunny, I stop for a moment and just look at the sun and just feel the warmth on my skin and just try to take a deep breath and engage in the moment, within nature, just to feel alive. (P756, male).

### Recommendations for future mindfulness interventions

#### Theme 1. Social support is generated during in-person meetings so in-person was the more preferred method of delivery

All of the students preferred in-person delivery of the course. Among the students who only took the class in-person (*n* = 4, Cohort 1) they felt as though the course would lose the supportive community by moving online; which was an important component for them.

To be able to go to a community where I can like get other insights and opinions and like share the experience with other people. So, I don’t think that the, I don’t think that doing it online would be very effective. Just because you don’t really get that personal like interaction you know? Like you don’t get that sense of community. (003, female).

Students (*n* = 11, Cohort 2) who, at week 5, were moved to online learning due to the coronavirus pandemic confirmed that this was their perception. They explained that the social support and sense of community they’d had in person was lost or reduced in their mind when the class was moved to digital delivery.

When you’re in the presence of others and you’re able to see everyone’s faces more clearly and assess how they’re breathing, how they’re acting if they’re fidgeting stuff like that. In person, you can see a lot more about someone’s body language and understand how they’re feeling and be able to empathize with them and the response of the instructor is a little bit better. It’s a little less, less poised when you’re in-person. And I think when you have that atmosphere being in-person, the just the raw body language that you get out of everything. Everyone gets a better experience. (223, female).

The class was also a safe physical space more conducive to practice for students and this seemed to be lost in digital delivery. One student (P001) from Cohort 1 explained that they believed it would be difficult for students to find protected space (e.g., quiet area, alone for two hours) to practice meditation and students (*n* = 3) that took the class digitally explained that they did find it difficult take the class in a space conducive to practice.

It would probably take away from the program if it was completely digital. And I just really value like having the like in-person type setting. I think that’s kind of what grounded me throughout this program and that’s what motivated me to continue with it in like setting goals. (P001, female).

Online is more difficult, especially like if you’re using zoom at home just finding a comfortable spot and I feel like you can’t really connect with everybody around you on webcam versus in-person. (P540, female).

#### Theme 2. A split class format would fit into schedules more effectively but could be difficult for those who commute

The majority (*n* = 13) preferred a split class approach. Students believed that if the class was delivered twice a week they would have more opportunities to have a space set aside for self-care each week even though they would be shorter sessions and that this would increase their frequency of practice because there would be less time between classes (e.g. 3 days instead of 1 week).

It just allows for people to attend rather than say we have a 2.5 hour class where that may cut into someone’s time elsewhere or like another class or like an appointment or just different things that people can have going on in a two and a half hour gap, rather than a one and a half hour gap. (P206, female).

Some indicated it would be easier to attend two shorter classes while others acknowledged that, for busy commuter students, meeting twice a week could be difficult. In fact, one student (P540) indicated that many of the students that signed up during recruitment but didn’t attend class chose to do so because the length of time (i.e., 2.5 hours) later in the day was a barrier. In addition, they suggested holding the class earlier in the day so that it could be incorporated into a free period rather than holding it at 6:00 PM after most classes were completed would make it easier to attend.

Having two classes a week might be a little, or two classes that are shorter and time might be a little bit easier and I think that also when you presented this course to some of the other people in my first year seminar class. I know a lot of people signed up for it on like the clipboard. But no, not a lot of people actually ended up going because some people knew that two and a half hours that they weren’t gonna be able to fit that into their schedules. (P540, male).

## Discussion

This study explored the experiences of undergraduates in a mindfulness-based program at a college of opportunity. Of the 16 students who participated, 43% were considered first-generation college students (*n* = 6) however in reviewing the data, comparing coding frequencies, number of references per participant, and discussing with content experts no notable differences by FGCS vs non-FGCS were identified. As a result, findings were presented for the overall sample rather then stratified by FGCS status. Overall, students found mindfulness useful and believed that mindfulness training enhanced stress management. They found it contributed to their well-being, even while having busy schedules, reduced the stress from commuting as well as the heavy responsibilities in balancing work/academic life, and the uncertainty of the ongoing global pandemic. Concerning intervention delivery, students stated that due to multiple responsibilities (e.g., pressures of commuting to class or balancing job/academic time schedules), they preferred in-person delivery, and that the intervention should be offered in split, shorter sessions in the morning or early afternoon.

Regarding the application and initiation of mindfulness, students reported that, over the 4-month period from beginning the intervention to completing the interview, they used mindfulness practices that could be easily incorporated into their daily routine (i.e., mindfully walking, eating mindfully, etc.) as opposed to those practices that required more accommodations and planning (e.g., longer sitting practice, body scans, etc.). It was revealed that these shorter, impromptu practices often enhanced the quality and attention paid to existing behaviors such as eating, engaging in physical activity, or studying. For some students, mindfulness also enhanced the enjoyment of activities and increased students’ motivation to engage in self-care. The most common application mindfulness practices were managing the wide array of stressors such as working, COVID-19, academic stress, and specific life situations (e.g., difficulties in living situation, ongoing mental health conditions, etc.). While engaging in mindfulness for stress management, students seemed to report becoming more resilient and able to recover faster from challenging situations which was especially important for students managing ongoing mental health diagnoses (e.g., anxiety, depression). Although many themes emerged, the process of stress management was perhaps explained most vividly Students described a temporal process by which mindfulness increased present-moment awareness allowing them to notice stressors and mentally ground themselves. After exercising this self-regulation, they were able to decide on a more effective, less reactive course of action. In many cases, those actions involved letting go of difficulties beyond their control as well as acting with mindfulness and self-care in situations in which they found they had higher levels of agency.

Previous results from the MBC program’s Stage 1 randomized controlled trial [25] offer consistencies as well as some clear contrasts with the current qualitative findings reported here. Qualitative analyses from the previous study were primarily focused on the acceptability and feasibility of the program specifically logistic considerations and intervention administration (i.e., clear understanding of course content, importance of the instructor, length of the course) [25]. During focus group discussions students in the previous Stage 1 trial did indicate similar applications of “in-the-moment” mindfulness practices for academic stress management and anxiety consistent with the current analyses [25]. They also stated they would prefer to have shorter, split classes that fit more effectively with a busy academic schedule consistent with the current study’s findings [25]. Results from the present analysis build on this previous work by providing more extensive details on the application of intervention components in students’ daily lives. In contrast to the previous study, in which use of intervention content was primarily focused on academic stress, students in the present study indicated the mindfulness practices were applied across multiple sources of stress including ongoing job responsibilities, academic stress, families and homelife, commuting to school, as was as the COVID-19 pandemic. This is a critical point of the present research: informal mindfulness practices and moment-to-moment stress management techniques extended to other domains of students’ lives (e.g., job, family, commuting, COVID-19, etc.) indicating that the intervention has the capacity to enhance well-being beyond university-specific stress among college students whose responsibilities necessitate a higher level of work/life balance. In addition, results from the present study indicate that stressors may not just have been reduced, as found in the previous work, but reframed. For example, when some students integrated the mindfulness practices into everyday activities that were previously stressful, such as studying or mental health related coping, they reported experiencing a shift in their relationship with the experience, in some cases enhancing resilience resources (i.e., self-care, acceptance) and in others reframing stressful activities into mindfulness practice opportunities.

The work here not only builds on previous research with the MBC program but offers important findings for a growing body of qualitative inquiry regarding students experience within mindfulness training more broadly. Qualitative explorations of MBPs and health among college students remain understudied and are limited to investigations conducted primarily in specific programs medical, nursing and other health singular academic programs limiting the transferability of results to mixed academic samples [24, 45–51]. However, some encouraging points should be noted: research has found that students believe mindfulness training enhances individual level resources (i.e., present moment awareness, attention control, self-regulation, self-compassion), increases their ability to balance stress and other academic priorities with purposeful action to enhance well-being (i.e., techniques to cognitively reframe when faced with academic induced anxiety and depression), and increases their self-reported empathy and communication skills in clinical training environments [24, 45, 48, 51, 52]. Additionally, in a 2020 systematic review of qualitative research to investigate students perceptions of MBPs found that across studies (k = 18) the main benefits reported were increased self-awareness, stress management, self-acceptance as well as academic focus while barriers to engagement included a lack of time for meditation outside of the program and uncertainty regarding MBPs impacts on health all of which are conclusions consistent with the findings from this study [23].

Student life has also changed substantially since the COVID-19 pandemic began and our work is part of a limited body of research that has qualitatively explored FGCS and non-FGCS experiences engaging in mindfulness training since the onset of the pandemic. Although FGCS experience singular stressors (e.g., initially navigating academia, potentially lack of academic preparation, lack of social support) we found no difference in qualitative findings regarding the application of mindfulness to cope with increasing stress and how students report this impacting their health and well-being [53]. The findings that FGCS and non-FGCS students described facing similar stressors (e.g., shifting from campus to home, commuting, classwork, etc.) is surprising but not unique. Recent studies show that although FGCS students were more likely to find it harder to complete academic tasks at home during COVID they were also more likely than non-FGCS students to seek out services and connect with support to overcome pandemic stress and anxiety [54, 55]. Taking this into account, it may not be surprising that FGCS from this study applied practices as regularly and to similar stressors when compared with non-FGCS peers. These findings indicate that as the frequency and strength of stressors (e.g., relocation, online learning, health specific anxiety) increase on both FGCS and non-FGCS as a result of the pandemic, group MBPs such as MBC present an effective, novel strategy of supporting mental health and sustaining social connection for students moving forward.

The analyses presented here are important as they not only build on growing bodies of work, but offer novel insights such as the role of mindfulness in enhancing resilience among students with diagnosed mental health conditions, vivid descriptions of students process of acceptance and letting go of pandemic stressors, and novel, detailed accounts of the integration of mindfulness into daily health behaviors. In addition, we present students’ experiences across a diverse student population (e.g., FGCS versus non-first generation, general student population, psychology students, part versus full-time, commuter versus on-campus residence) and provide both pragmatic and psychosocial insights into how students apply mindfulness interventions across personal (i.e., family), professional (i.e., work life balance) and academic domains to enhance their well-being. In addition, previous work has minimally investigated students’ perceptions regarding the most effective delivery modalities and logistic considerations for mindfulness interventions among undergraduate students navigating multiple, complex, and interrelated stressors (e.g., commuting to class, living off campus, work/life balance). This work provides important information for university administrators as they select and customize group-based therapeutics, such as MBPs, for student health and well-being by offering insights into how students receive and apply these interventions as well as the most effective delivery modalities given the necessity to balance time, professional, and personal responsibilities for health and well-being. Lastly, this research represents one of the few didactic qualitative inquiries into students’ application of mindfulness practices during the COVID-19 pandemic.

### Limitations and future directions

A limitation of these data is that they were drawn from a relatively small sample of participants (*n* = 16) from one academic institution which lowers transferability compared to if the study was from multiple institutions. However, discussions between coders as well as within the larger team meetings suggested that saturation was achieved, and this sample is distinct given many participants were first generation, commuting students with full-time jobs. A second limitation is although the proportion of FGCS was greater than our previous research, the number of students was six students, thereby potentially limiting the diversity of experiences. This could have contributed to the lack of differences observed between groups in coding discussions and thematic development. A targeted recruitment strategy towards FGCS coupled with specific inclusion criteria regarding FGCS status may have yielded a higher number of FGCS. A higher number of FGCS may have yielded a broader range of lived experience for qualitative analyses. The low recruitment could explain the lack of difference observed thematically between FGCS and non-FGCS. In addition, specific interview questions regarding the FGCS status may have elicited more direct feedback regarding mindfulness among FGCS participants (e.g., “Tell me a bit about what it’s like being the first person in your family to go to college”, etc.). Lastly, during analysis participants were not asked to review findings or transcripts for inconsistencies and did not provide feedback on the findings presented. Allowing for transcript review could have elicited additional information or allowed participants to correct any responses they feel need to be revised.

Despite limitations, this study provides valuable insight into the utility of customized MBPs for undergraduate students. Future research should build off qualitative findings investigating efficacy and effectiveness utilizing mixed method study designs (e.g., exploratory sequential study designs) among larger sample sizes and include multi-site research strategies across multiple academic institutions. In addition, future research should prioritize access of the intervention to diverse groups of students including larger percentages of racially, ethnically, socioeconomically, and culturally diverse students to investigate for comprehensively the utility and continuing customizations required for MBPs.

## Conclusion

This study reveals several important findings. First, these data suggest that more practical and efficiently incorporated mindfulness-based strategies are of more value than mindfulness-based strategies that require a high degree of time set aside since time is a limited resource for students with a broader array of personal and professional responsibilities. Also, these data suggest that briefer modules and more flexibility to complete weekly tasks and components of the intervention are a better fit for students building on our previous qualitative work. Although the research is preliminary, it provides important insights into the potential for mindfulness training to support students as the prevalence of mental health conditions (e.g., anxiety, depression, etc.) continues to increase nationally and offers clear recommendations from students for universities to effectively offer MBPs to support their well-being.

## Supplementary Information

**Additional file 1: S1 File.** Mindfulness-College Qualitative Codebook: File contains the qualitative codebook with operationalized coding definitions and coding frequencies both total number for each code as well as number of data sources containing the referenced code.

**Additional file 2: S2 File.** Semi-Structured Interview Protocol.
