# Supplementary material for: Students experiences of an 8-week mindfulness-based intervention at a college of opportunity: a qualitative investigation of the mindfulness-based college program
Source: BMC Public Health. 2022 Dec 13;22:2331. doi: 10.1186/s12889-022-14775-5 (PMC9745283; doi:10.1186/s12889-022-14775-5)
Supplement: Supplementary file 1 — Additional file 1: S1 File. Mindfulness-College Qualitative Codebook: File contains the qualitative codebook with operationalized coding definitions and coding frequencies both total number for each code as well as number of data sources containing the referenced code. [file 12889_2022_14775_MOESM1_ESM.docx]

| **MB-College Interview Guide**   \| Welcome and introduction   - Thank you for sharing your thoughts on MB-College - Introductions - Why and How   - We are trying to improve the intervention   - Discussing your opinions on the different activities   - Please share your point of view   - No wrong answers   - We equally welcome positive and negative feedback - Suggestions   - - Speak up     - Audio recording of interviews discussions one person speaks at a time     - Any report that we write about what we hear today will not be associated with your identity     - Tendency for some people to be comfortable speaking up more than others. It’s important to us to hear from everyone today. So, I may ask you to share if I haven’t hear from you. Or I may ask you to let others share if you are sharing a lot. - What to expect   - My role is to listen, facilitate discussion between you and move us along 5 questions.   - In the interest of time, I may have to move the discussion along so that we can cover all 5 questions   - Let’s begin \|  \| \| --- \| --- \| \| **Question 1**: What was most helpful about this course, and why?  **Suggested Probes:**  - Was there something you learned in the course that was helpful?  - Could you give an example from your experience?  - Was there a specific activity that you found most helpful? \|  \| \| **Question 2.** After going through this mindfulness intervention, what is your understanding of how it works to improve your health?  **Suggested Probes:**  - Could you give an example from your experience?  - Have you seen changes in your performance?  - If yes what do you attribute to causing these changes? \|  \| \| **Question 3**. We want to make this intervention better. You have been through it once. How do you think we can make it better?  **Suggested Probes:**  - Where there any activities (e.g. breakout groups) you think could be improved?  - Were there any improvement on the space for the class?  - How was the timing for the course? (e.g. length, time of day, time distribution of course activities)  - Is there anything you think we can do to improve on the communications? (e.g. email, phone, text) \|  \| \| **Question 4.** Every instructor can improve. How can this instructor improve?  **Suggested Probes:**  - Was the communication clear?  - Did you feel like you were understood? \|  \| \| **Intervention Delivery Questions:**  Thank you so much. We just have a few more questions. In the next series of questions, I’ll be asking for your thoughts on the time and delivery of the intervention.   - The class was 2.5 hours. Do you think that that was enough time, or could it have been shorter, for example 2 hours or longer, 3 hours? - The all-day retreat you were asked to participate in was 7.5 hours. Did you feel like this could have been shorter, for example 6 hours or longer for example 8 hours? - The course was delivered once per week. One question we have is whether it would be easier for students to attend two shorter (e.g. 1.5 hour) courses over the week as opposed to 1 longer course. What are your thoughts on this?   The program was delivered in person. We are considering offering it in an online form like a digital class via Zoom. What are your thoughts on digital delivery of this program? \|  \| \| Pass out survey/Email Qualtrics link to digital interview participants.  “I’m going to hand out a quick survey, which I’d like for you to fill out.” \|  \| \| Discussion summary  Is there anything we should have talked about, but didn’t? \|  \| |
| --- | --- | --- | --- | --- | --- | --- | --- | --- | --- | --- | --- | --- | --- | --- | --- | --- |
