# Supplementary material for: Students experiences of an 8-week mindfulness-based intervention at a college of opportunity: a qualitative investigation of the mindfulness-based college program
Source: BMC Public Health. 2022 Dec 13;22:2331. doi: 10.1186/s12889-022-14775-5 (PMC9745283; doi:10.1186/s12889-022-14775-5)
Supplement: Supplementary file 2 — Additional file 2: S2 File. Semi-Structured Interview Protocol. [file 12889_2022_14775_MOESM2_ESM.docx]

## Supplementary Material 2: MB-College at RIC Codebook

| **Aims/**  ***Themes*** | | Total # Transcripts w/ Theme | Total # Of Codes per Theme |
| --- | --- | --- | --- |
| **Aim 1:** Exploring the Application of Mindfulness Practices in Daily Life | |  |  |
| ***Theme 1:*** *The most used practices are those that could be integrated into daily life.* | |  |  |
| *Mindfulness Practices Outside of Class* | Situations where participants suggested they took "mindful action" or engaged in daily practices with awareness outside of class, for example, mindful eating.  **Note:** Mindful walking, daily practice and mindful eating are specific sub-codes of practice outside of class. | 15 | 65 |
| *Mindful Walking* | Participants describing formal and informal walking meditations.  **Note:** This was a sub-code of “Mindfulness Practices Outside of Class”, Walking and eating mindfully were the two most described practices and necessitated their own sub-coding to review. | 8 | 20 |
| *Mindful Eating* | Participants used the practice of eating mindfully in their life either as a new practice or to enhance an existing routine.  **Note:** This was a sub-code of “Mindfulness Practices Outside of Class”, Walking and eating mindfully were the two most described practices and necessitated their own sub-coding to review. | 10 | 25 |
| ***Theme 2.*** *Students often applied mindfulness to existing behaviors and activities.* | | | |
| *Mindfulness Enhances Existing Routine* | Participant explains why they gravitated towards certain mindful practices and incorporated them into their existing routines. | 15 | 75 |
| *Daily Practice* | Details of the practice’s participants stated they used daily as part of their existing routine.  **Note:** Daily practice speaks specifically to those practices that participants engage in daily as part of an existing routine. For participants who say the like a particular meditation without speaking to how regularly they engage in its coding would be under the parent code of “Mindfulness practices outside of class.” | 13 | 38 |
| ***Theme 3.*** *Mindfulness increased students’ engagement activities motivated by self-care* | | | |
| *Engaging in Self-Care* | Participants reporting moments when they engaged in behaviors for self-care to improve health and wellbeing during/after the course.  **Note:** This does not include descriptions of what participants engaged in prior to starting the course. | 11 | 30 |
| **Aim 2: Exploring students’ perception of mindfulness practices effect on health and well-being** | | | |
| ***Theme 1.*** *Mindfulness was used most often to cope with an array of stressors.* | | | |
| *Overall Stress Management* | Situations where participants used mindfulness practices to work with stressful situations. Examples could include, breathing through anxiety or practicing compassion with a difficult family member. The important piece is the application of a mindfulness practice for a stressful situation. | 15 | 68 |
| *Stressful Living Situation* | Situations where participants used mindfulness to work with difficult living situations. | 1 | 3 |
| *Anxiety* | Situations where participants used mindfulness practices to work with anxiety. | 7 | 20 |
| *Self-Kindness* | Participants being "warm and understanding toward ourselves when we suffer, fail, or feel inadequate, rather than ignoring our pain or flagellating ourselves with self-criticism." | 6 | 13 |
| ***Theme 2.*** *Mindfulness practices help to handle COVID-19 specific* | | | |
| *COVID-19 Related Stress* | Participants making note of the pandemic and how they used mindfulness to navigate the complications that introduces to their lives. Most often pertaining to stress management.  **Note**: There are relationships between coding specific to living situations, stress management, and anxiety with the COVID Related Stress. However, this code is specific to stress that was either caused by or exacerbated by the pandemic. As this study took place both before and at the onset of the pandemic not all stress described is specific to the COVID era. | 8 | 32 |
| ***Theme 3.*** *Mindfulness enhanced stress coping by increasing the ability to acceptance of the present moment and let go of the experience to move forward.* | | | |
| *Letting Go* | Situations where participants acknowledge their present moment experience and take action to release/relinquish what they are experiencing (e.g., thoughts, emotions, social situations, physical challenges, etc.).  **Note:** There appears to be a relationship between letting go and acceptance. Letting go has been reported in conjunction with moments of acceptance. | 10 | 20 |
| *Acceptance* | Participants detailed situations when they stopped resisting their present moment experiences/situations (e.g., mental states, living situations, or social situations, etc.) and began to open to the fact that "this is how it is." . | 8 | 26 |
| *Lower Self-Criticism* | Situations where participants decreased their self-criticism because of the program. This could include points when they did not fulfill a goal, or something did not go as planned.  **Note:** Coding indicated a relationship between decreased self-criticism and their ability to be accepting. | 3 | 7 |
| *Non-judgement* | Discussions of becoming less critical or judgmental of present moment experiences.  **Note:** A relationship seemed to be indicated for non-judgment and acceptance when coding was reviewed such that many stories of acceptance involved being non-judgmental with a situation. | 4 | 13 |
| ***Theme 4.*** *Students also believe mindfulness enhanced resilience, especially for those with existing mental health conditions.* | | | |
| *Resilience* | Participants reporting on recovering more effectively after a stressful or difficult situation. Could also relate to courage or perseverance. | 9 | 15 |
| *Mindfulness Enhances Recovery* | Mindfulness facilitates recovery from addictive habits (e.g., food, substance use, gambling, shopping) | 2 | 7 |
| *Increased Patience* | Participant reports of increasing patience with themselves, circumstances, and others after participating in the program. | 3 | 6 |
| ***Theme 5.*** *Mindfulness was critical for grounding themselves during difficult experiences as well as enhancing the value of positive experiences.* | | | |
| *Grounding* | Situations where participants used skills developed in class to feel stabilized. | 16 | 54 |
| *Mind~Body Awareness* | Participants descriptions of a mind/body connection. | 10 | 24 |
| *Gratitude* | Expressing appreciation for the program or other aspects of their lives. | 4 | 5 |
| **Aim 3: Recommendations for Future Mindfulness Interventions** | | | |
| ***Theme 1.*** *Social support is generated during in-person meetings so in-person more preferred method of delivery.* | | | |
| *Class Critiques* | Coding only for participant’s direct responses to question 4 of the interview guide: “We want to make the intervention better. You have been through it once. How do you think we can make it better?” as well as the interviewer’s responses. Do not use for course critiques that may occur in other questions. | 14 | 28 |
| *Instructor Comments* | Coding only for participant’s direct responses to question 5 of the interview guide: "Every instructor can improve. How can this instructor improve?" as well at the interviewer’s responses. Do not use for instructor feedback that may occur in other questions, please file under relevant context code. Example: Participant Z states in question 1 that the instructor’s clear communication was most memorable. | 14 | 28 |
| ***Theme 2.*** *A split class format would fit into schedules more effectively but could be difficult for those who commute.* | | | |
| *Course Structure Delivery* | Data related to the time, date, online versus in-person, and other delivery suggestions/recommendations | 15 | 29 |
